# Integrative Metabolomic and Transcriptomic Analysis Provides Novel Insights into the Effects of SO_2_ on the Postharvest Quality of ‘Munage’ Table Grapes

**DOI:** 10.3390/foods13213494

**Published:** 2024-10-31

**Authors:** Zhenliang Mou, Yuyao Yuan, Wei Wei, Yating Zhao, Bin Wu, Jianye Chen

**Affiliations:** 1 Guangdong Provincial Key Laboratory of Postharvest Science of Fruits and Vegetables, Engineering Research Center of Southern Horticultural Products Preservation, Ministry of Education, College of Horticulture, South China Agricultural University, Guangzhou 510642, China; mouzhenliang@stu.scau.edu.cn (Z.M.); weiwei@scau.edu.cn (W.W.); 2 Xinjiang Key Laboratory of Processing and Preservation of Agricultural Products, Institute of Agro-Products Storage and Processing, Xinjiang Academy of Agricultural Sciences, Urumqi 830091, China; yuan1357991@sina.com; 3 College of Food Science and Pharmacy, Xinjiang Agricultural University, Urumqi 830052, China; zhaoyating@scau.edu.cn

**Keywords:** SO_2_, table grapes, metabolomics, transcriptomics, flavonoid metabolism

## Abstract

Postharvest grapes exhibit a limited shelf life due to susceptibility to rot and deterioration, significantly reducing their nutritional and economic value. Sulfur dioxide (SO_2_) is a widely recognized preservative for extending grape storage life. This study performed a detailed analysis of ‘Munage’ table grapes treated with SO_2_ fumigation, employing transcriptomic and metabolomic approaches. Results indicate that SO_2_ fumigation significantly extends the shelf life of grapes, as demonstrated by improved visual quality, reduced decay rates, and increased fruit firmness. We identified 309 differentially accumulated metabolites (DAMs) and 1906 differentially expressed genes (DEGs), including 135 transcription factors (TFs). Both DEGs and DAMs showed significant enrichment of flavonoid-related metabolism compared with the control, and the relative content of four flavonoid metabolites (Wogonin-7-O-glucuronide, Acacetin-7-O-glucuronide, Apigenin-7-O-glucuronide, and Baicalein 7-O-glucuronide) were significantly increased in grapes upon SO_2_ treatment, suggesting that SO_2_ treatment had a substantial regulatory effect on grape flavonoid metabolism. Importantly, we constructed complex regulatory networks by screening key enzyme genes (e.g., *PAL*, *4CLs*, *CHS*, *CHI2*, and *UGT88F3*) related to the metabolism of target flavonoid, as well as potential regulatory transcription factors (TFs). Overall, our findings offer new insights into the regulatory mechanisms by which SO_2_ maintains the postharvest quality of table grapes.

## 1. Introduction

Table grapes are widely recognized as a significant cash crop and are characterized as non-climacteric fruits enriched with beneficial compounds including vitamins, flavonoids, and micronutrients [1]. The Munage grape (*Vitis vinifera* L. cv. Munage.), indigenous to the Xinjiang Autonomous Region of China, is extensively cultivated owing to its unique flavor, robust resistance, and late ripening properties [2]. However, the quality of harvested grapes declines rapidly due to active physiological metabolism and susceptibility to pathogen infection, which contribute to issues such as softening, loss of weight, rachis browning, and decay. These factors markedly diminish the economic value and export potential of the table grapes [3].

In recent years, researchers have developed innovative technologies for postharvest preservation of grapes, including chitosan [4], nitric oxide [5], and melatonin [6]. Despite these advancements, sulfur dioxide (SO_2_) has been the dominant method for commercial storage of table grapes for over 90 years, underscoring its irreplaceable effects and critical role in maintaining grape quality during storage and transportation. Current research on SO_2_ preservation primarily investigates its physiological impacts and mechanisms. Recent studies indicate that SO_2_ treatment effectively reduces decay and preserves the postharvest appearance of various fruits, including blueberries [7], cherries [8] and pomegranate [9]. In grape, SO_2_ is crucial for enhancing disease resistance [10], triggering defense responses [11], and supporting energy metabolism [2]. Additionally, SO_2_-fumigated grapes show reduced decay due to the activation of the AsA-GSH cycle and mitigation of oxidative damage [12]. SO_2_ treatment has been shown to maintain higher firmness and preserve cell wall integrity in postharvest grapes, which can be attributed to the inhibition of ethylene signaling [13]. In addition, SO_2_ has been suggested to alter the accumulation of metabolites, thereby positively affecting the postharvest quality of grapes. For instance, SO_2_ treatment increased the content of phenolic compounds by activating flavonoid-related genes, inhibiting the grape rachis browning [14]. Along with the activation of secondary metabolic pathways, SO_2_-fumigated grapes accumulated higher levels of total phenols, flavonoids, and lignans, resulting in improved resistance [10]. Furthermore, the synthesis of stilbenes and some phenolic acids is suppressed by SO_2_ fumigation, which helps reduce decay by modulating the phenylpropane metabolic pathway [11]. However, the specific compounds and comprehensive networks influenced by SO_2_ in maintaining postharvest grape quality remain largely unexplored.

Omics technologies have been widely utilized to identify the pivotal genes and functional metabolites involved in regulating the quality of horticultural products. Transcriptome analysis provides expression profiles of the grape senescence process [15]. Recent studies have identified pathogenesis-related genes and cell wall degradation-related genes in postharvest grapes by transcriptomics technology [13]. Techniques such as RNA sequencing (RNA-seq) and ultra-high performance liquid chromatography tandem mass spectrometry (UHPLC-MS/MS) have elucidated mechanisms underlying grape color development [1]. Additionally, the impact of melatonin [6], chitosan [4], and UV-C [3] on grape quality has been explored through transcriptomics and metabolomics, revealing variation in metabolite accumulation across different grape varieties and treatments. However, the combined use of transcriptomics and metabolomics to investigate the effects of SO_2_ on postharvest grape quality has not yet been implemented.

In this study, we elucidated the effects of SO_2_ on the quality of ‘Munage’ grapes via RNA-seq and untargeted metabolomic analyses. Through a comprehensive assessment of differentially expressed genes (DEGs) and differentially accumulated metabolites (DAMs), we identified key regulatory pathways and target metabolites of SO_2_ treatment on postharvest quality maintenance in grapes. In addition, by weighted gene co-expression network analysis (WGCNA), we constructed co-expression networks concerning 13 structural genes and 47 potential regulatory TFs. The results offer novel insights into the regulatory pathways of SO_2_ on grape quality maintenance.

## 2. Materials and Methods

### 2.1. Plant Materials and Samples

‘Munage’ table grapes, achieving commercial maturity at a total soluble solids (TSS) content of 19.7%, were harvested from a vineyard proximate to Atushi County, Xinjiang, China. Fruit selection criteria included the absence of physical damage and uniformity in cluster size and color. Approximately 500 g of grapes were randomly allocated into boxes (dimensions: 19 cm × 15 cm × 8 cm), with a total of 120 boxes prepared. These were then randomly assigned into two treatment groups, each comprising three biological replicates. Building on our previous experiments [16], we optimized the treatment conditions as follows: (1) SO_2_ treatment group, the boxes were placed in a fumigation box and SO_2_ gas was injected into it by controlling the released velocity with a flow meter to attain a SO_2_ concentration of 2000 μL L^−1^. The fumigation box was placed at 10 °C and the SO_2_ fumigation of the grapes was sustained for 2 h. (2) For the control group, the grapes were placed in the same condition without SO_2_ gas. After the treatments, all grapes were stored at 10 °C with 85% relative humidity for 20 days. Samples consisting of 60 fruits were randomly collected from each treatment group every 5 days and subsequently stored at −80 °C.

### 2.2. Determination of Physicochemical Indices of Grapes

At each sampling time, the firmness of 30 berries (from 5 different bunches) were measured using a firmness tester (GY-4, Handpi, Leqing, Zhejiang, China). For decay evaluation, 40 berries per treatment were examined from each biological replicate. Decay incidence was determined based on the area of spoilage, following a previously reported method [17].

### 2.3. Transcriptome Sequencing and Analysis

Based on our preliminary test, the control group began to lose commercial value at 10 °C after exceeding 10 days. Therefore, grape tissues (10 g) from both the control and SO_2_ treatments at 0, 10, 15, and 20 d were selected for RNA-seq and comprehensive untargeted metabolite analysis, with three biological replicates at each sampling point: CK-0d, CK-10d, CK-15d, CK-20d, SO_2_-0d, SO_2_-10d, SO_2_-15d, and SO_2_-20d. With the assistance of Metware Biotechnology Co., Ltd (Wuhan, China), the total RNA of the grapes was extracted by ethanol precipitation and CTAB-PBIOZOL. Subsequently, a Qubit fluorescence quantifier and a Qsep400 high-throughput biofragment analyzer were used for identifying and quantifying the total RNA. The libraries passing the check were sequenced in Illumina, and clean reads were obtained using fastp (v0.23.2) from the original sequence information by removing reads with adapters, ploy-N, and low-quality reads (Q ≤ 20 bases account for more than 50% of the total read length). Clean reads were aligned using Hisat2 (v2.2.1) base on the reference genome (GCF_000003745.3, https://www.ncbi.nlm.nih.gov/datasets/genome). Gene expression levels and read mapping were quantified using FPKM (fragments per kilobase million) values and featureCounts (v2.0.3), respectively [18,19]. Principal component analysis (PCA) and Pearson correlation coefficients (PCC) were conducted to assess overall differences using the R package. Genes with absolute fold change ≥ 2 and the parameter of FDR < 0.05 were screened by DESeq2 (v1.22.1) as DEGs [20]. For the comparison of DEGs, KEGG enrichment analysis wascarried out.

### 2.4. Metabolite Extraction and Analysis

Grape samples were lyophilized and ground using a lyophilizer (Scientz-100F) and a grinder (MM 400, Retsch, Haan, Germany). Approximately 50 mg of the resulting powder was immersed in 1200 μL of pre-cooled 70% methanol solution. The mixture was vortexed six times, once every 30 min for 30 s, followed by centrifugation at 12,000 rpm for 3 min. The supernatant obtained by filtration (0.22 μm membrane) was used as the injection solution for UPLC-MS/MS analysis.

The extract was analyzed using a UPLC-ESI-MS/MS system, which primarily included ultra-performance liquid chromatography (UPLC, ExionLC™ AD, Framingham, MA, USA) and tandem mass spectrometry (MS/MS). Analytical conditions were as follows: UPLC, column, Agilent SB-C 18 (2.1 mm × 100 mm, 1.8 µm); solvent system, pure water with 0.1% formic acid (A), acetonitrile with 0.1% formic acid (B). The gradient program began with 95% A and 5% B, transitioning to 5% A and 95% B over 9 min, held for 1 min, and then returned to 95% A and 5% B within 1.1 min, maintained for an additional 2.9 min. The flow rate was 0.35 mL/min, and the column temperature was maintained at 40 °C. The injection volume was 2 μL. The effluent underwent further analysis by an ESI-triple quadrupole-linear ion trap (QTRAP)-MS, with both linear ion trap and triple quadrupole (QQQ) scans being performed. ESI source operation parameters included a source temperature of 550 °C, ion spray voltage (IS) of 5500 V (positive) and −4500 V (negative), with ion source gas I (GSI), gas II (GSII), and curtain gas (CUR) set at 50, 60, and 25 psi, respectively.

The data were processed using Analyst (version 1.6.3). The metabolites were identified based on self-built MetWare database and quantified using scheduled multiple reaction monitoring (MRM). Missing values were supplemented with one-fifth of the minimum values, and the coefficient of variation (CV) was calculated for the quality control (QC) samples; only those metabolites with a CV value less than 0.5 considered as confidently identified. A VIP ≥ 1 and a fold change ≥2 or ≤0.5 were considered DAMs. Annotated DAMs were mapped to KEGG Pathway and MetMap databases to infer the putative functions of DAMs using the Metware Cloud. Then, the DAMs were clustered by *k*-means using the R software (R 4.1.2).

### 2.5. Association Analysis of the Metabolome and Transcriptome

To integrate transcriptomic (FPKM > 1) and metabolomic data, unsigned co-expression networks were constructed using WGCNA (v1.69). After discarding low-expressed or non-varying genes filtered by median absolute deviation, a total of 16,051 genes were used to obtain co-expression network modules, with the final power of 10, minModuleSize of 30, and a branch merge height of 0.4. The module eigengene value was calculated for assessing the correlations between modules and SO_2_-regulated key DAMs in the process of grape storage. Co-expression networks, derived from Pearson correlation analyses between structural genes and TFs, were visualized using Cytoscape (v3.8.0).

### 2.6. qRT-PCR Analysis

To verify the RNA-seq data, the expression of nine flavonoid-related genes, including those encoding TFs in brown module, were validated by quantitative real-time PCR (qRT-PCR). The cDNA required for qRT-PCR was prepared as described by Mou et al. [21]. Specific primers were designed in primer 5 and are shown in Table S1. The sequence of Actin-7 (LOC100232866) was chosen as an endogenous reference [12].

### 2.7. Statistical Analysis

The data of physiological characteristics from three biological replicates were analyzed by GraphPad Prism 9.4 software (Graph Pad Software, San Diego, CA, USA). In the significant characteristics at *p* < 0.05 or below, mean values of control and SO_2_ treatment were compared on days 0, 5, 10, 15, and 20 using Student’s *t*-test at significance level *p* < 0.05 or 0.01.

## 3. Results

### 3.1. Effect of SO_2_ Treatment on the Quality Indices in Grapes

**Figure 1:**
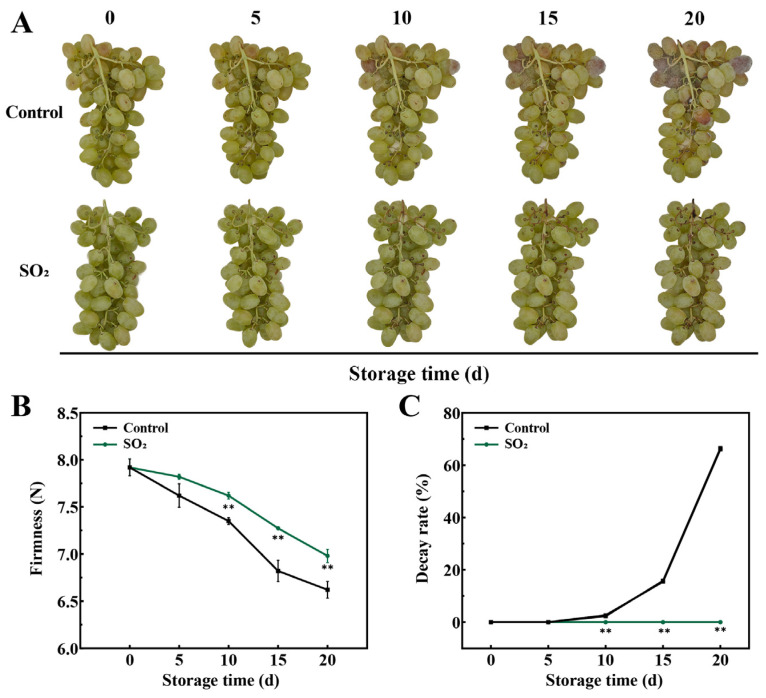
The SO_2_ treatment maintains the appearance and postharvest quality of ‘Munage’ table grapes during storage at 10 °C for 20 d. (**A**) Alterations in visual appearance of control and SO_2_-treated grapes. (**B**,**C**) Comparison of firmness and decay rate in ‘Munage’ grapes under control and SO_2_ treatment. The data in (**B**,**C**) represent the average plus or minus standard error of three biological replicates. Asterisks indicate statistical significance between control and SO_2_-treated grapes, with ** indicating *p* < 0.01.

The appearance of ‘Munage’ table grapes from both treatment groups remained largely unchanged in the initial 5 days (Figure 1A). However, browning of the berry skins, pulp shrinkage, and slight rotting symptoms were observed in the control grapes by day 10. Further observations revealed the presence of white and black hyphae in the control grapes on day 10, which extensively covered the berries by the end of the storage period. In contrast, no visible hyphae were observed on the SO_2_-treated grapes throughout the entire storage period. Our previous study showed that SO_2_ application could maintain berry firmness, total soluble solids (TSS), and titratable acidity (TA) contents, while reducing decay, weight loss, and abscission rate [2,12]. Therefore, in this study, we measured only berry firmness and decay rate. As shown in Figure 1B, berry firmness in the control grapes decreased progressively during storage. Compared to the control group, the SO_2_ treatment slowed down grape softening and significantly inhibited the decrease in firmness on days 10, 15, and 20 (*p* < 0.05). As expected, decay rate (Figure 1C) in the control group increased steadily with storage time, reaching 66.12%, which was considerably higher than that in the SO_2_-treated grapes. Collectively, SO_2_ treatment played a positive role in retarding the berries’ decay and softening, which maintained the appearance and quality of grapes.

### 3.2. Transcriptomic Analysis

#### 3.2.1. General Description of Transcriptomic Data

**Figure 2:**
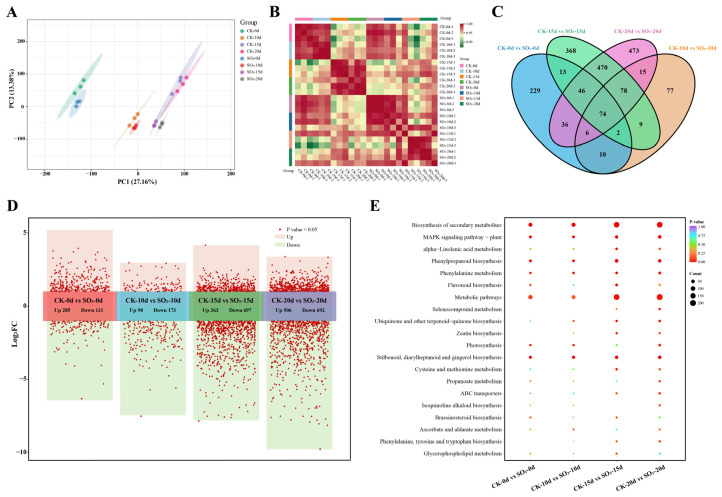
Overview of ‘Munage’ table grapes transcriptome with control and SO_2_ treatment. (**A**) PCA score plot profiles and (**B**) correlation heat map from transcriptomic data. (**C**) Venn diagram presenting the differentially expressed genes (DEGs) in CK-0d vs. SO_2_-0d, CK-10d vs. SO_2_-10d, CK-15d vs. SO_2_-15d, and CK-20d vs. SO_2_-20d comparisons. (**D**) Volcano plots of up-regulated and down-regulated genes between control and SO_2_-treated group. (**E**) KEGG enrichment pathways of DEGs from different comparison groups.

Due to the significant differences observed in phenotypic and quality indices between control and SO_2_ treatment groups, RNA-seq analysis was performed on 24 samples of ‘Munage’ grape to investigate gene expression changes. The transcriptome sequencing obtained a total of 161.61 GB clean reads, averaging approximately 6 GB from each sample, with Q30 ranging from 94.54% to 95.33% in grape samples. The GC content ranged from 46.54% to 47.17%, and the error rate was 0.02%, ensuring high-quality transcriptome sequencing results. Between 91.50% and 94.63% of clean reads were successfully mapped to the chosen reference genome, directly reflecting a high utilization of the transcriptome (Table S2). Additionally, PCA analysis revealed notable differences in unigene expression between the treatment groups (Figure 2A). PC1 accounted for 27.16% of the total variance, effectively separating samples based on different time points, particularly distinguishing the grape samples at 0 d. At the PC2 level, CK-15d was evidently separable from SO_2_-15d, and CK-20d from SO_2_-20d. Consistent with the PCA, the intersample correlation heatmap demonstrated high intragroup sample correlation among biological replicates, with PCC between 0.932 and 0.997 (Figure 2B).

#### 3.2.2. Analysis of DEGs in Grapes Under SO_2_ Treatment

All samples were classified into four comparison groups, and the number of DEGs, as well as the overlap among each comparison group, was illustrated by Venn diagram (Figure 2C) based on DEG filtering criteria. A total of 1906 DEGs were detected, including 285, 98, 363, and 506 up-regulated genes and 131, 173, 697, and 692 down-regulated genes obtained in the CK-0d vs. SO_2_-0d, CK-10d vs. SO_2_-10d, CK-15d vs. SO_2_-15d, and CK-20d vs. SO_2_-20d, respectively (Figure 2D). Additionally, the DEGs from four comparison groups were annotated with KEGG pathways. Enrichment analysis revealed that these DEGs were primarily involved in four metabolic pathways: phenylpropanoid biosynthesis (map00940), phenylalanine metabolism (map00360), flavonoid biosynthesis (map00941), MAPK signaling pathway-plant (map04016), and stilbenoid, diarylheptanoid, and gingerol biosynthesis (map00945) (Figure 2E). Notably, three of the significantly enriched pathways were related to flavonoid metabolism, indicating differential expression of genes associated with flavonoid metabolism and biosynthesis between the control and SO_2_ treatments.

**Figure 3:**
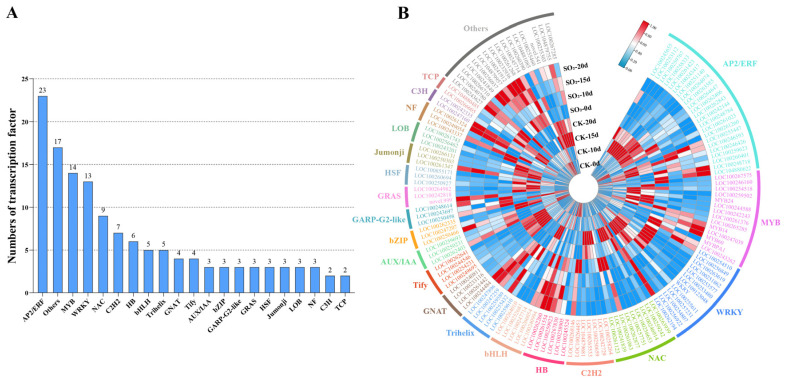
Transcription factors (TFs) involved in SO_2_-regulated quality in postharvest grapes. (**A**) Statistics of differentially expressed TFs from different families. (**B**) Heat map showing the expression trends of differentially expressed TFs.

Gene expression is tightly regulated by transcription factors (TFs). To explore TFs potentially involved in SO_2_-regulated grape quality, we identified 135 TFs from 21 TF families (Figure 3A), mainly consisting of 23 AP2/ERF TFs, 14 MYB TFs, 13 WRKY TFs, 9 NAC TFs, 7 C2H2 TFs, and 6 HB TFs. Heatmap analysis was performed to illustrate their expression patterns (Figure 3B). The results showed that the expression of most AP2/ERF, WRKY, and NAC family TFs was repressed under SO_2_ treatment, whereas the expression of certain MYB, bHLH, and HB family members was significantly up-regulated during grape storage. These results suggest that multiple SO_2_-mediated TFs are cooperatively involved in maintaining the quality of grapes.

### 3.3. Metabolomic Analysis

#### 3.3.1. Analysis of DAMs in Grape Under SO_2_ Treatment

**Figure 4:**
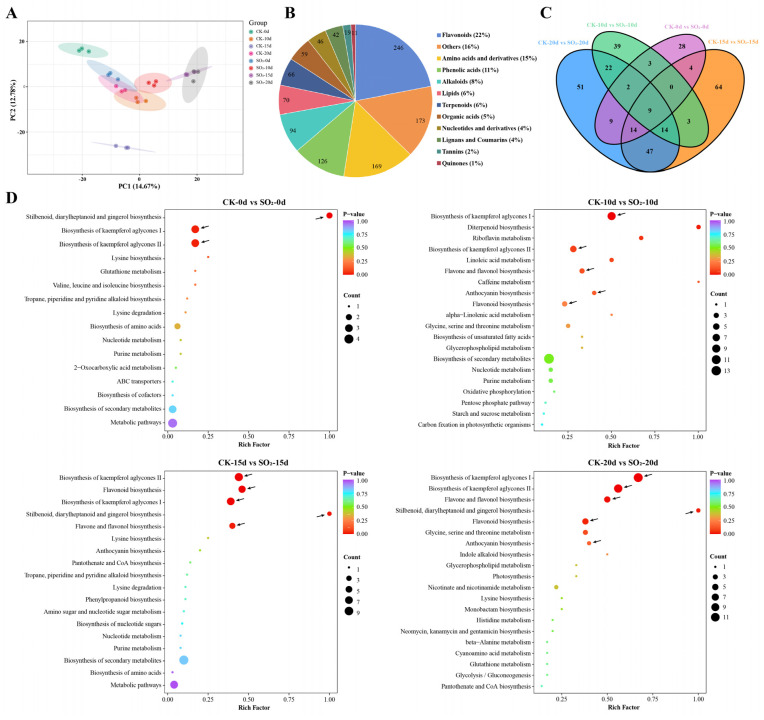
Overview of ‘Munage’ table grapes metabolome with control and SO_2_ treatment. (**A**) PCA of control and SO_2_-treated group from metabolomics data. (**B**) Statistics of the types and amounts of identified metabolites. (**C**) Venn diagram presenting the differentially accumulated metabolites (DAMs) in the CK-0d vs. SO_2_-0d, CK-10d vs. SO_2_-10d, CK-15d vs. SO_2_-15d, and CK-20d vs. SO_2_-20d comparisons. (**D**) KEGG enrichment pathways of DAMs from different comparison groups.

In this study, an untargeted metabolomics approach (LC-MS) was employed to identify DAMs in grape samples subjected to SO_2_ treatment, aiming to elucidate the regulatory mechanism by which SO_2_ maintains grape quality. A score scatterplot illustrating the distinct separation between samples is presented in Figure 4A. A total of 1122 metabolites were successfully identified across 24 grape samples, which were classified into 12 subclasses: flavonoids (246), others (173), amino acids and derivatives (169), phenolic acids (126), alkaloids (94), lipids (70), terpenoids (66), organic acids (59), nucleotides and derivatives (46), lignans and coumarins (42), tannins (19), and quinones (11) (Figure 4B). Venn diagram analysis revealed 309 DAMs across the four comparison groups (Figure 4C; Table S3). Specifically, 69, 92, 155, and 168 DAMs were detected in the comparisons of CK-0d vs. SO_2_-0d, CK-10d vs. SO_2_-10d, CK-15d vs. SO_2_-15d, and CK-20d vs. SO_2_-20d, respectively. To comprehensively understand the functions of these DAMs, metabolic pathway enrichment analysis was conducted. As shown in Figure 4D, six metabolic pathways were mainly enriched, including biosynthesis of kaempferol aglycones I (MetMap113), kaempferol aglycones II (MetMap114), flavonoid biosynthesis (ko00941), flavone and flavonol biosynthesis (ko00944), stilbenoid, diarylheptanoid, and gingerol biosynthesis (ko00945), and anthocyanin biosynthesis (ko00942). Overall, both the metabolome and transcriptome exhibit significant enrichment of flavonoid-related metabolism, implying that the accumulation of flavonoids may play a crucial role in the ability of SO_2_ treatment to maintain grape quality during storage.

#### 3.3.2. Analysis of Key Metabolites in Grape Under SO_2_ Treatment

**Figure 5:**
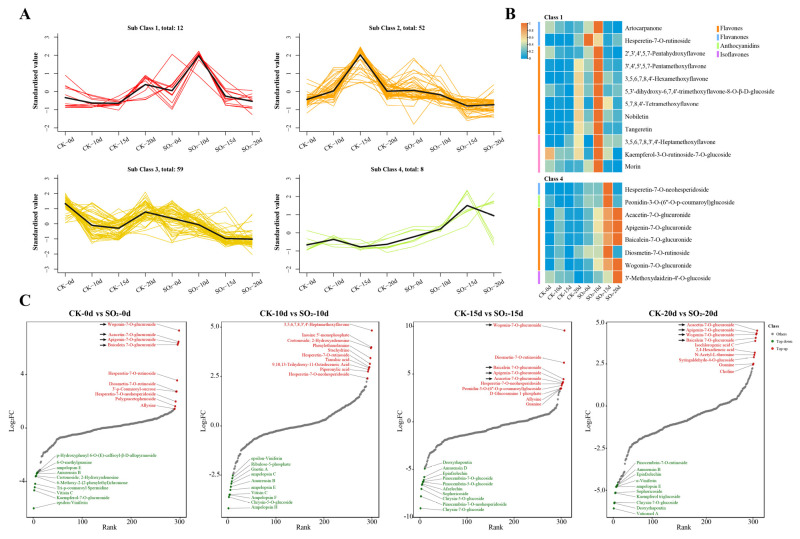
Analysis of DAMs associated with SO_2_ maintaining grape quality. (**A**) *k*-means clustering analysis of differentially accumulated flavonoid metabolites. (**B**) Heat map of the DAMs in class 1 and class 4. (**C**) Up-regulated and down-regulated DAMs in different comparison group (TOP10).

To obtain the accumulation dynamics of flavonoid metabolites throughout the entire storage period, 131 differentially accumulated flavonoid metabolites were divided into 4 distinct major clusters by *k*-means clustering analysis (Figure 5A). We focused on classes 1 and 4, where the relative content of flavonoids was significantly higher in SO_2_ treatment than the control group. The heatmap further verified the observed changes in relative metabolite contents of class 1 and class 4, which were consistent with the *k*-means results (Figure 5B). The analysis showed that 12 flavonoids were clustered in class 1, including 7 flavones (3,5,6,7,8,4′-Hexamethoxyflavone, 2′,3′,4′,5,7-Pentahydroxyflavone, 3′,4′,5′,5,7-Pentamethoxyflavone, Tangeretin, 5,3′-dihydroxy-6,7,4′-trimethoxyflavone-8-O-β-D-glucoside, Nobiletin, and 5,7,8,4′-Tetramethoxyflavone), 3 flavonols (3,5,6,7,8,3′,4′-Heptamethoxyflavone, Kaempferol-3-O-rutinoside-7-O-glucoside, and Morin), and 2 flavanones (Artocarpanone and Hesperetin-7-O-rutinoside). Class 4 included eight flavonoids, consisting of five flavones (Acacetin-7-O-glucuronide, Apigenin-7-O-glucuronide, Baicalein 7-O-glucuronide, Diosmetin-7-O-rutinoside and Wogonin-7-O-glucuronide), one flavanone (Hesperetin-7-O-neohesperidoside), one anthocyanidin (Peonidin-3-O-(6′′-O-p-coumaroyl) glucoside), and one isoflavone (3′-Methoxydaidzin-4′-O-glucoside). In addition, we screened the top up-regulated and down-regulated DAMs across four comparison groups (Figure 5C). Among them, the four up-regulated flavones (Wogonin-7-O-glucuronide, Acacetin-7-O-glucuronide, Apigenin-7-O-glucuronide, and Baicalein 7-O-glucuronide) were found in all comparisons of CK-0d vs. SO_2_-0d, CK-15d vs. SO_2_-15d, and CK-20d vs. SO_2_-20d, while they also were categorized in group 4. These data suggest that SO_2_ treatment induces a higher accumulation of the four flavones in grapes at all stages of storage, suggesting they may serve as key metabolites in maintaining grape quality under SO_2_ treatment.

### 3.4. Co-Expression Network Analysis Associated with Key Flavonoids

**Figure 6:**
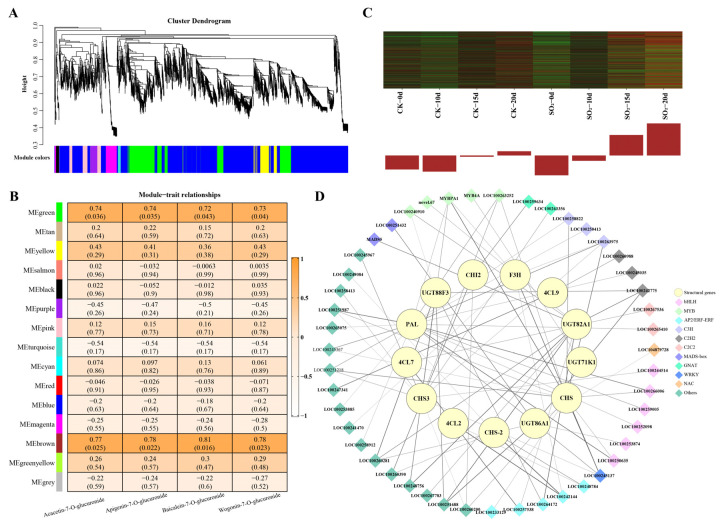
Correlation networks constructed by weighted gene co-expression network analysis (WGCNA). (**A**) Hierarchical clustering tree of 16,051 genes (with FPKM ≥ 1) by WGCNA. (**B**) Module–trait associations, with the columns and rows representing specific flavonoid compounds and modules, respectively. Correlation coefficients are displayed via a color scale, with *p*-values in parentheses. (**C**) Heat map and common expression patterns according to the FPKM of each gene in the brown module. (**D**) Co-expression regulatory network of flavonoid-related genes and TFs in the brown module. Yellow circles denote the structural genes involved in flavonoid biosynthesis. Differently colored diamonds represent TFs from various families, whose expressions were significantly positively correlated with the identified genes (PCC > 0.85). Connecting lines are drawn based on the correlation coefficients of the nodes, with darker colors indicating stronger correlations. Abbreviations are defined as follows: PAL, phenylalanine ammonia-lyase; CHS, chalcone synthase; 4CL, 4-coumarate-CoA ligas; CHI, chalcone-flavonone isomerase; F3H, flavanone 3-dioxygenase; UGT, UDP-glycosyltransferase.

To construct gene regulatory networks for key metabolites, 16,051 genes (FPKM > 1) were entered into the WGCNA analysis, identifying fifteen modules, with the module sizes from 48 (cyan) to 5046 (turquoise) (Figure 6A). In the module–trait relationships, the brown module showed the highest positive correlations with the relative content of key metabolites (Wogonin-7-O-glucuronide, Acacetin-7-O-glucuronide, Apigenin-7-O-glucuronide, and Baicalein-7-O-glucuronide) (r > 0.75, 0 < *p* < 0.03) (Figure 6B). Heatmaps and eigengene histograms revealed that the gene expressions from the brown module were up-regulated by SO_2_ treatment (Figure 6C), especially on days 15 and 20, aligning with the accumulation pattern of the target metabolites. These results indicate that the brown module is closely associated with key metabolites and can be considered a critical module for further analysis.

In the brown module, we identified 13 flavonoid-metabolizing genes, including 4 *UDP-glycosyltransferase* (*UGT*), 3 *chalcone synthase* (*CHS*), 3 *4-coumarate-CoA ligase* (*4CL*), 1 *flavanone 3-dioxygenase* (*F3H*), 1 *phenylalanine ammonia-lyase* (*PAL*), and 1 *chalcone-flavonone isomerase* (*CHI*) (Figure 6D; Table S4). Based on Pearson’s correlation coefficients, 47 TFs were highly positively correlated with these structural genes mentioned above (PCC > 0.85), mainly consisting of 6 bHLH TFs, 5 MYB TFs, 5 AP2/ERF-ERF TFs, 3 C3H TFs, 3 C2H2 TFs, 2 C2C2 TFs, and MADS-box TFs (Figure 6D; Tables S4 and S5). We infer that these TFs potentially participate in the accumulation of key metabolites by regulating the expression of these structural genes.

To validate the RNA-Seq results, nine genes from the brown module were randomly selected for qRT-PCR analysis, including three flavonoid-related structural genes (*UGT82A1*, *4CL7*, *CHS3*) and six flavonoid related TFs (*LOC100245372*, *LOC100257538*, *LOC100260284*, *LOC100264000*, *LOC100264303*, *LOC100267536*) (Figure S1). We observed that the expression level of the genes showed strong concordance with the transcriptome data. Additionally, *UGT82A1, CHS3*, and all screened TFs were significantly highly expressed in SO_2_-treated grape on day 7 (*p* < 0.05). These findings confirm the reliability of the RNA-seq results and the identified putative genes.

## 4. Discussion

Grapes are thin-skinned berries with high water content, making them extremely susceptible to quality deterioration during storage, primarily manifesting as postharvest decay. To mitigate this issue, the use of SO_2_ has become a common practice in the grape industry, although its safety remains a topic of debate. Despite these concerns, numerous studies have confirmed that SO_2_ is a widely used preservative to control decay and extend the shelf life of grapes [12]. In this study, the decay incidence of the control samples increased continuously after 5 days of storage (Figure 1C), accompanied by extensive hyphae growth and browning of berry skins (Figure 1A). SO_2_ treatment efficiently reduced decay and pathogen infection in grape, as well as prevented the reduction of berry firmness (Figure 1). These findings are consistent with reports across various grape varieties, indicating that SO_2_ is highly effective in preserving postharvest grape quality [11,14].

Secondary metabolites have garnered increasing attention due to their roles in plant growth, ripening, and defense [22]. Flavonoids, the largest class of secondary metabolites, are synthesized through the phenylpropanoid pathway and can be mainly classified into flavonols, flavones, isoflavones, flavanones, and anthocyanins [23]. Grapes are particularly rich in flavonoids, which significantly affect appearance, nutritive value, flavor, and antioxidant properties, thereby influencing grape quality and commercial value [24]. In this study, we identified 246 flavonoids, with more than half showing differential accumulation. Integrated metabolomic and transcriptomic data showed that the majority of DAMs and DEGs were mapped in phenylpropanoid and flavonoid biosynthesis (Figure 2E and Figure 4D). Similar results have been reported in previous studies, where SO_2_ treatment activated flavonoid metabolism in table grapes [13,15]. Furthermore, the content of flavonoid metabolites, including Wogonin-7-O-glucuronide, Acacetin-7-O-glucuronide, Apigenin-7-O-glucuronide, and Baicalein 7-O-glucuronide significantly increased in grapes treated with SO_2_ (Figure 5B,C). Numerous studies have shown that the Wogonin-7-O-glucuronide and Baicalein 7-O-glucuronide, as major flavones in the Chinese herbal medicine *Scutellaria baicalensis* Georgi, possess antioxidant, antibacterial, and antiviral bioactivities [25,26]. Apigenin-7-O-glucuronide was identified as an antioxidant active component in *Salvia* [27]. Thus, we speculate that SO_2_-evoked accumulation of these flavones can prevent attacks by pathogenic bacteria, resist senescence-induced oxidative stress, and maintain the quality of table grapes. In addition to flavonoids, another branch in the phenylpropanoid pathway is the synthesis of stilbenes [28]. Our study also observed the involvement of DAMs and DEGs in stilbenoid metabolism (Figure 2E and Figure 4D), with SO_2_-treated grapes showing lower stilbene levels (Figure 5C). Although stilbene has a role in disease resistance [29], our result was consistent with recent omics analysis showing that SO_2_ reduced the synthesis of stilbene and effectively alleviated berry decay [11]. A possible explanation for the reduced stilbene content could be species differences or the shunting effect of the phenylpropanoid pathway. Additionally, we found that other flavonoids, such as flavonols and anthocyanins, were down-regulated by SO_2_ treatment. This finding aligns closely with recent studies that melatonin treatment inhibits the expression of genes related to anthocyanin biosynthesis, which helps alleviate chilling injury in plums during storage [30]. Similarly, H_2_O_2_ treatment led to a reduction in the accumulation of 11 flavonoids and 5 flavonols, effectively delaying postharvest senescence in broccoli [31].

WGCNA has been demonstrated as an effective method for identifying potentially relevant genes [32]. In this study, WGCNA and PCC analysis were employed to construct co-expression networks. The results revealed a significant correlation between the brown module and the target flavones, including Wogonin-7-O-glucuronide, Acacetin-7-O-glucuronide, Apigenin-7-O-glucuronide, and Baicalein 7-O-glucuronide (Figure 6B). Additionally, the expression of genes within this module was activated by SO_2_ treatment (Figure 6C), suggesting that candidate genes involved in SO_2_-regulated flavonoid metabolism and the postharvest quality of grape are located in the brown module. Previous research has comprehensively elucidated that a series of key structural genes play crucial roles in flavonoid biosynthesis in some model plants [33,34]. The phenylpropanoid metabolic pathway, an early step in flavonoid biosynthesis, converts phenylalanine to p-coumaroyl CoA through the action of several enzymes, such as PAL, cinnamate 4-hydroxylase (C4H), and 4CL [35]. Subsequently, p-coumaroyl CoA is condensed and isomerized by CHS and CHI, ultimately being catalyzed to naringenin, a core precursor for flavonoid synthesis that can be converted into various flavonoid subclasses [36]. In *Scutellaria baicalensis*, PAL, C4H, 4CL, and CHS have been validated as key genes in the synthesis of flavones such as Baicalein 7-O-glucuronide and Wogonin-7-O-glucuronide [37]. UGTs are involved in the glycosylation of flavones [38]. In this study, 13 flavonoid-metabolizing genes were identified in the brown module (Figure 6D), including *PAL*, *4CL2*, *4CL9*, *4CL7*, *CHS*, *CHS-2*, *CHS3*, *CHI2*, *UGT88F3*, *UGT82A1*, *UGT86A1*, *UGT71K1*, and *F3H*. Based on the involvement of these structural genes in flavonoid biosynthesis, it is strongly hypothesized that these 13 hub genes are key contributors to SO_2_-regulated flavonoid biosynthesis. In addition, the flavone synthase (FNS), a rate-limiting enzyme of flavones biosynthesis [39], was not detected in this study, which may suggest post-translational regulation or its absence in the RNA-seq data.

An increasing number of studies have shown that flavonoid biosynthesis is largely coordinated by TFs, especially from MYB, bHLH, WRKY, and MADS-box proteins families [40]. For instance, overexpression of *MYB5a* up-regulates the expression of *CHS*, *F3H*, and *CHI*, leading to increased anthocyanin and flavonol content in tobacco [41]. In addition, *VvMYBPA2* participates in anthocyanin biosynthesis to combat biotic stress in grapevine (*Vitis vinifera*) [42]. In bananas, *MabHLH363* is involve in the flavonoid biosynthesis responding to stress by regulating the transcription of *MaUGTs* [43]. The role of the MYB-bHLH-WDR complex in regulating flavonoid biosynthesis has been well established [44]. A previous study indicated that many up-regulated MYB and bHLH family TFs were found in SO_2_-fumigated grapes [13]. In this study, 47 TFs, primarily from nine families including bHLH, MYB, ERF, MADS-box, and WRKY, were strongly correlated with the identified flavonoid-metabolizing genes (Figure 6D; Tables S4 and S5). Among them, TFs from the bHLH and MYB families were the most prevalent, comprising six bHLH TFs (*LOC100244514*, *LOC100250635*, *LOC100252098*, *LOC100253874*, *LOC100259005*, and *LOC100266006*) and five MYB TFs (*LOC100240910*, *LOC100263252*, *MYB4A*, and *MYBPA1, novel.67*), suggesting that bHLH and MYB families play important roles in regulating flavonoid metabolism. Other known TF families involved in flavonoid biosynthesis were also detected, including five ERF TFs (*LOC100233129*, *LOC100242144*, *LOC100248784*, *LOC100257538*, and *LOC100264172*), two MADS-box TFs (*MADS5* and *LOC100251432*), and one WRKY TF (*LOC100245137*). Recent studies indicate that ERF TFs mediate flavonoid biosynthesis in navel orange [45], lichi [46], and cabbage [47]. In buckwheat, the overexpression of *FDMADS28* increased rutin content and enhanced resistance [48]. Additionally, Wang et al. revealed the function of *MdWRKY11* in flavonoid biosynthesis in apple (*Malus domestica* Borkh) [49]. Apart from the above TF families, there were another 29 TFs belonging to many families, such as C3H, C2H2, C2C2, and GANT, indicating that SO_2_ might mediate multiple TFs to regulate candidate structural genes, thereby accumulating specific flavonoids that are beneficial for maintaining postharvest quality in grapes. While qRT-PCR has confirmed that the identified structural genes and TFs are up-regulated by SO_2_ (Figure S1), further molecular experiments are necessary to determine the specific roles of these genes in flavonoid biosynthesis.

## 5. Conclusions

In the present study, SO_2_ application inhibited berry decay and softening, and thus could effectively maintain the quality of postharvest grapes. Through integrative metabolomic and transcriptomic analysis, we focused on the flavonoid metabolism pathway and identified the four most significantly altered flavones in SO_2_-fumigated grapes, such as Baicalein 7-O-glucuronide and Wogonin-7-O-glucuronide, which may contribute to the preservation of grape quality. Furthermore, we revealed gene co-expression modules using WGCNA, and identified 47 regulatory TFs and 13 downstream genes (*PAL*, *4CL2*, *4CL9*, *4CL7*, *CHS*, *CHS-2*, *CHS3*, *CHI2*, *UGT88F3*, *UGT82A1*, *UGT86A1*, and *UGT71K1*), which may be involved in mediating key flavonoids induced by SO_2_. Taken together, these findings reveal the underlying regulatory mechanism for SO_2_ retardation of grape quality deterioration through the accumulation of specific flavonoid compounds.

## Data Availability

The original contributions presented in this study are included in the article/Supplementary Materials. Further inquiries can be directed to the corresponding authors.

## Supplementary Materials

The following supporting information can be downloaded at: https://www.mdpi.com/article/10.3390/foods13213494/s1, Figure S1: The qRT-PCR analysis of transcripts in brown modules. Table S1: Primer sequences used for qRT-PCR in the present study. Table S2. Overview of RNA-Seq results and transcriptome assembly. Table S3: Identified differentially accumulated metabolites detected in 24 samples of ‘Munage’ grapes during storage. Table S4. Correlation network file of SO_2_-regulated the quality of grape in brown module. Table S5. Pearson correlation coefficient of flavonoid-related genes with TFs in co-expression regulatory network.

## References

[1] Ju Y.L., Wang W.N., Yue X.F., Xue W., Zhang Y.L., Fang Y.L. (2023). Integrated metabolomic and transcriptomic analysis reveals the mechanism underlying the accumulation of anthocyanins and other flavonoids in the flesh and skin of teinturier grapes. Plant Physiol. Biochem..

[2] Zhang Z., Xing S.J., Yuan Y.Y., Zheng Y.G., Tian Q.M., Wu B., Wei J. (2024). Sulfur dioxide fumigation promotes GABA accumulation and energy metabolism to delay quality deterioration in postharvest table grapes. Postharvest Biol. Technol..

[3] Zhang K.K., Li W.P., Ju Y.L., Wang X.H., Sun X.Y., Fang Y.L., Chen K.Q. (2021). Transcriptomic and metabolomic basis of short- and long-term post-harvest UV-C application in regulating grape berry quality development. Foods.

[4] Zhang Z.B., Zhao P.C., Zhang P.A., Su L.Y., Jia H.R., Wei X.K., Fang J.G., Jia H.F. (2020). Integrative transcriptomics and metabolomics data exploring the effect of chitosan on postharvest grape resistance to *Botrytis cinerea*. Postharvest Biol. Technol..

[5] Wu Z.H., Dong C.H., Wei J., Guo L.M., Meng Y.N., Wu B., Chen J.L. (2021). A transcriptional study of the effects of nitric oxide on rachis browning in table grapes cv. Thompson seedless. Postharvest Biol. Technol..

[6] Ma W.Y., Xu L.L., Gao S.W., Lyu X., Cao X.L., Yao Y.X. (2021). Melatonin alters the secondary metabolite profile of grape berry skin by promoting *VvMYB14*-mediated ethylene biosynthesis. Hortic. Res..

[7] Saito S., Obenland D., Xiao C.L. (2020). Influence of sulfur dioxide-emitting polyethylene packaging on blueberry decay and quality during extended storage. Postharvest Biol. Technol..

[8] Yang Y.T., Gong Y., Gao Y.F., Huang J.Y., Xu M., Xiong B. (2020). Study on the preservation effect of propolis on sweet cherry. Earth Environ. Sci..

[9] Ali Q., Dogan A., Erkan M. (2024). Sulfur dioxide generating pads containing different concentrations of sodium metabisulfite maintains postharvest quality of ‘hicaznar’ pomegranate. Sci. Hortic..

[10] Xue M.Z., Yi H.L. (2017). Induction of disease resistance providing new insight into sulfur dioxide preservation in *Vitis vinifera* L.. Sci. Hortic..

[11] Zhang J., Xie L.J., Wang H.J., Zhou S.H., Zhu Z.Q., Xie T.L., Zhou Y.M., Li W., Pang L.T., Sun J. (2024). Metabolome and transcriptome analyses provide insight into the effect of 1-MCP and SO_2_ preservatives on the synthesis and regulation of phenols in ‘Shine Muscat’ storage grapes. LWT.

[12] Zhang Z., Wu Z.H., Yuan Y.Y., Zhang J.X., Wei J., Wu B. (2022). Sulfur dioxide mitigates oxidative damage by modulating hydrogen peroxide homeostasis in postharvest table grapes. Postharvest Biol. Technol..

[13] Yan D.M., Yi H.L. (2024). Transcriptome analysis provides insights into preservation mechanism of postharvest muscat hamburg grapes treated with SO_2_. Sci. Hortic..

[14] Li Z.B., Chen S.Q., Qi M., Yang M.Y., Yuan H.M., Xu Y.Q., Huang J., Li D., Zhou W., Yuan Y.Y. (2023). Inhibition of postharvest rachis browning of table grapes by sulfur dioxide: Evidence from phenolic metabolism and sulfur assimilation. Postharvest Biol. Technol..

[15] Xue M.Z., Yi H.L., Wang H. (2018). Identification of miRNAs involved in SO_2_ preservation in *Vitis vinifera* L. by deep sequencing. Environ. Exp. Bot..

[16] Zhang Z., Wei J., Wang M., Zhang J., Wu B. (2022). Induced sulfur metabolism by sulfur dioxide maintains postharvest quality of ‘thompson seedless’ grape through increasing sulfite content. J. Sci. Food Agric..

[17] Zhang Z., Xu J., Chen Y., Wei J., Wu B. (2019). Nitric oxide treatment maintains postharvest quality of table grapes by mitigation of oxidative damage. Postharvest Biol. Technol..

[18] Liao Y., Smyth G.K., Shi W. (2014). featureCounts: An efficient general purpose program for assigning sequence reads to genomic features. Bioinformatics.

[19] Yang Z.C., Lin M.H., Yang X.Z., Wu D., Chen K.S. (2023). Comprehensive analysis of transcriptome and metabolome provides insights into the stress response mechanisms of apple fruit to postharvest impact damage. Food Chem. Mol. Sci..

[20] Love M.I., Huber W., Anders S. (2014). Moderated estimation of fold change and dispersion for RNA-Seq data with DESeq2. Genome Biol..

[21] Mou Z.L., Wang L., Zeng Z.X., Su X.G., Ji S.J., Shan W., Kuang J.F., Lu W.J., Chen Y.L., Zhao Y.T. (2023). Metabolomics integrated with transcriptomics unveil the regulatory pathways of modified atmosphere packaging–maintained leaf quality of chinese flowering cabbage. Food Chem..

[22] Song M.Y., Wang L., Zhang Y.T., Wang Q.G., Han X., Yang Q., Zhang J.H., Tong Z.K. (2023). Temporospatial pattern of flavonoid metabolites and potential regulatory pathway of *PbMYB211*-coordinated kaempferol-3-O-rhamnoside biosynthesis in *Phoebe bournei*. Plant Physiol. Biochem..

[23] Guven H., Arici A., Simsek O. (2019). Flavonoids in our foods: A short review. J. Basic Clin. Health Sci..

[24] Han S.A., Xie H., Wang M., Zhang J.G., Xu Y.H., Zhu X.H., Caikasimu A., Zhou X.W., Mai S.L., Pan M.Q. (2023). Transcriptome and metabolome reveal the effects of three canopy types on the flavonoids and phenolic acids in ‘Merlot’ (*Vitis vinifera* L.) berry pericarp. Food Res. Int..

[25] Zhao Q., Zhang Y., Wang G., Hill L., Weng J.K., Chen X.Y., Xue H.X., Martin C. (2016). A specialized flavone biosynthetic pathway has evolved in the medicinal plant, *Scutellaria baicalensis*. Sci. Adv..

[26] Zhao Q., Yang J., Cui M.Y., Liu J., Fang Y.M., Yan M.X., Qiu W.Q., Shang H.W., Xu Z.C., Yidiresi R. (2019). The reference genome sequence of *Scutellaria baicalensis* provides insights into the evolution of wogonin biosynthesis. Mol. Plant..

[27] Shojaeifard Z., Hemmateenejad B., Jassbi A.R. (2021). Chemometrics-based LC-UV-ESIMS analyses of 50 *Salvia* species for detecting their antioxidant constituents. J. Pharm. Biomed. Anal..

[28] Donnez D., Jeandet P., Clément C., Courot E. (2009). Bioproduction of resveratrol and stilbene derivatives by plant cells and microorganisms. Trends Biotechnol..

[29] Rivière C., Pawlus A.D., Mérillon J. (2012). Natural stilbenoids: Distribution in the plant kingdom and chemotaxonomic interest in vitaceae. Nat. Prod. Rep..

[30] Xu R.R., Wang L.M., Li K.X., Cao J.K., Zhao Z.L. (2022). Integrative transcriptomic and metabolomic alterations unravel the effect of melatonin on mitigating postharvest chilling injury upon plum (cv. Friar) fruit. Postharvest Biol. Technol..

[31] Wang Y.Q., Zhang Y.X., Guo Y.Y., Ji N.N., Chen Y., Sun Y.P., Wang Z.L., Guan L.X., Guo P.C. (2024). Integrated transcriptomic and metabolomic analysis reveals the effects and potential mechanism of hydrogen peroxide on pigment metabolism in postharvest broccoli. J. Food Sci..

[32] Zhang B., Horvath S. (2005). A general framework for weighted gene co-expression network analysis. Stat. Appl. Genet. Mol. Biol..

[33] Saito K., Yonekura-Sakakibara K., Nakabayashi R., Higashi Y., Yamazaki M., Tohge T., Fernie A.R. (2013). The flavonoid biosynthetic pathway in arabidopsis: Structural and genetic diversity. Plant Physiol. Biochem..

[34] Andersen J.R., Zein I., Wenzel G., Darnhofer B., Eder J., Ouzunova M., Lübberstedt T. (2008). Characterization of phenylpropanoid pathway genes within european maize (*Zea mays* L.) inbreds. BMC Plant Biol..

[35] Kim J.I., Hidalgo-Shrestha C., Bonawitz N.D., Franke R.B., Chapple C. (2021). Spatio-temporal control of phenylpropanoid biosynthesis by inducible complementation of a cinnamate 4-hydroxylase mutant. J. Exp. Bot..

[36] Yonekura-Sakakibara K., Higashi Y., Nakabayashi R. (2019). The origin and evolution of plant flavonoid metabolism. Front. Plant Sci..

[37] Cheng L., Han M., Yang L.M., Yang L., Sun Z., Zhang T. (2018). Changes in the physiological characteristics and baicalin biosynthesis metabolism of *Scutellaria baicalensis* Georgi under drought stress. Ind. Crop. Prod..

[38] Jiang Y.F.Y., Ji X.Y., Duan L.X., Ye P., Yang J.F., Zhan R.T., Chen W.W., Ma D.M. (2019). Gene mining and identification of a flavone synthase II involved in flavones biosynthesis by transcriptomic analysis and targeted flavonoid profiling in *Chrysanthemum indicum* L.. Ind. Crop. Prod..

[39] Wu J., Wang X.C., Liu Y., Du H., Shu Q.Y., Su S., Wang L.J., Li S.S., Wang L.S. (2016). Flavone synthases from *Lonicera japonica* and *L. macranthoides* reveal differential flavone accumulation. Sci. Rep..

[40] Zhang S., Yang J., Li H.Q., Chiang V.L., Fu Y.J. (2021). Cooperative regulation of flavonoid and lignin biosynthesis in plants. CRC Crit. Rev. Plant Sci..

[41] Deluc L., Barrieu F., Marchive C., Lauvergeat V., Decendit A., Richard T., Carde J.P., Mérillon J.M., Hamdi S. (2006). Characterization of a grapevine R2R3-MYB transcription factor that regulates the phenylpropanoid pathway. Plant Physiol..

[42] Terrier N., Torregrosa L., Ageorges A., Vialet S., Verriès C., Cheynier V., Romieu C. (2009). Ectopic expression of VvMybPA2 promotes proanthocyanidin biosynthesis in grapevine and suggests additional targets in the pathway. Plant Physiol..

[43] Song W., Lin S.Q., Yin Q., Liu T.H., Gan L.Z., Qi J.J., Yang Y.Y., Wei W., Shan W., Kuang J.F. (2024). A multi-omics approach reveals low temperature inhibition of flavones and flavonols accumulation in postharvest bananas via downregulation of MabHLH363. Postharvest Biol. Technol..

[44] Xu W.J., Dubos C., Lepiniec L. (2015). Transcriptional control of flavonoid biosynthesis by MYB–bHLH–WDR complexes. Trends Plant Sci..

[45] Wan H.L., Liu Y.H., Wang T.T., Jiang P., Wen W.W., Nie J.Y. (2023). Combined transcriptomic and metabolomic analyses identifies *CsERF003*, a citrus ERF transcription factor, as flavonoid activator. Plant Sci..

[46] Zhuo M.G., Wang T.Y., Huang X.M., Hu G.B., Zhou B.Y., Wang H.C., Abbas F. (2024). ERF transcription factors govern anthocyanin biosynthesis in litchi pericarp by modulating the expression of anthocyanin biosynthesis genes. Sci. Hortic..

[47] Yue L.Q., Kang Y.Y., Zhong M., Kang D.J., Zhao P.Y., Chai X.R., Yang X. (2023). Melatonin delays postharvest senescence through suppressing the inhibition of BrERF2/BrERF109 on flavonoid biosynthesis in flowering chinese cabbage. Int. J. Mol. Sci..

[48] Liu Y., Guan C.N., Chen Y.Y., Shi Y.L., Long O., Lin H., Zhang K.X., Zhou M.L. (2024). Evolutionary analysis of MADS-box genes in buckwheat species and functional study of *FdMADS28* in flavonoid metabolism. Plant Physiol. Biochem..

[49] Wang N., Liu W.J., Zhang T.L., Jiang S.H., Xu H.F., Wang Y.C., Zhang Z.Y., Wang C.Z., Chen X.S. (2018). Transcriptomic analysis of red-fleshed apples reveals the novel role of MdWRKY11 in flavonoid and anthocyanin biosynthesis. J. Agric. Food Chem..

