# Supplementary figures and images for: Integrative Metabolomic and Transcriptomic Analysis Provides Novel Insights into the Effects of SO2 on the Postharvest Quality of ‘Munage’ Table Grapes
Source: Foods. 2024 Oct 31;13(21):3494. doi: 10.3390/foods13213494 (PMC11545366; doi:10.3390/foods13213494)

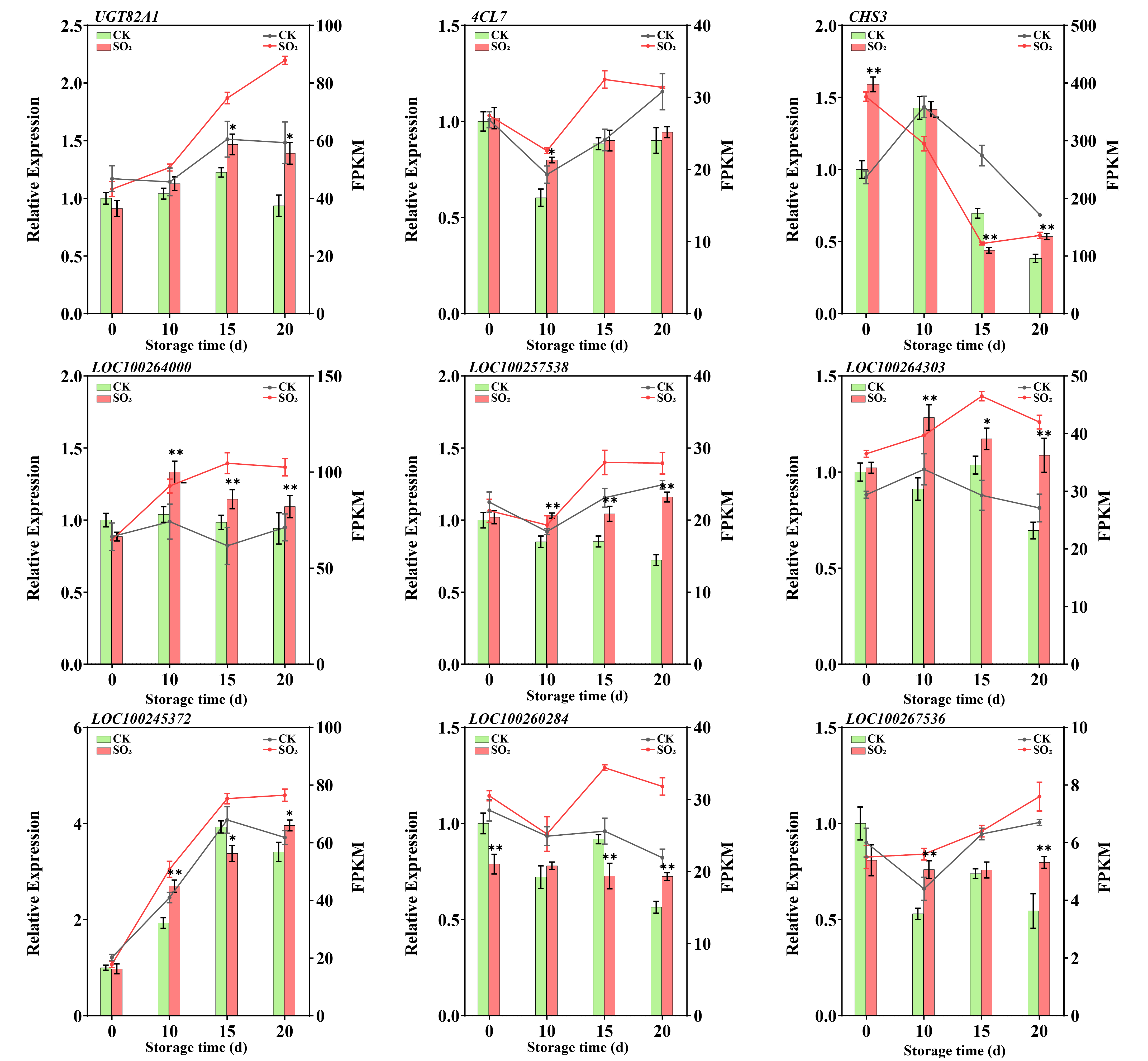

Supplement: Supplementary file 1 [file foods-13-03494-s001.zip › Figure S1.tif]
